# Temperature-Dependent Recombinase-Based Genetic Circuits

**DOI:** 10.3390/ijms262412055

**Published:** 2025-12-15

**Authors:** Marc Gonzalez-Colell, Mariana Gomes del Castillo, Marta Palau Gauthier, Javier Macia

**Affiliations:** Synthetic Biology for Biomedical Applications Lab, Department of Medicine and Life Sciences, Universitat Pompeu Fabra, Biomedical Research Park, 08003 Barcelona, Spain; marc.gonzalezi@upf.edu (M.G.-C.); marianaalexandra.gomesdel@upf.edu (M.G.d.C.); marta.palaui@upf.edu (M.P.G.)

**Keywords:** temperature-dependent, regulation, recombinase, circuits, RNA thermometers, synthetic biology, cellular patterning

## Abstract

Temperature offers a simple yet powerful signal to program cellular behavior. Here, we engineered and characterized a set of temperature-dependent genetic circuits that integrate RNA thermometers with site-specific DNA recombinases to achieve precise, irreversible control of gene expression. Using the serine recombinase Bxb1 placed under the control of the *Salmonella* FourU RNA thermometer, we demonstrate how promoter strength critically defines thermal sensitivity: weak promoters’ activity clears ON/OFF transitions, while strong promoters lead to continuous, quasi-temperature-independent recombination. Furthermore, temperature pulse duration and growth phase of cell culture were found to modulate recombination efficiency, providing additional layers of control. We illustrate the potential of this framework through proof-of-concept applications, including (i) the generation of spatial expression patterns on 2D surfaces via localized heating, (ii) a paper-based device capable of recording temperature gradients as stable genetic outputs, and (iii) a temperature-triggered lysis system for controlled cellular release. Together, these results establish temperature-regulated recombinase circuits as versatile and robust tools for programmable, spatially resolved, and irreversible control of gene expression, paving the way for new applications in synthetic biology, biosensing, and bioproduction.

## 1. Introduction

Temperature serves as a versatile and dynamic input signal to precisely orchestrate engineered cellular functions, enabling the control of engineered microorganisms across a wide range of applications [1,2,3,4]. Because temperature is a noninvasive and deeply penetrant signal, it can be employed both globally and with spatial specificity [5]. Alterations in temperature can therefore regulate gene expression in various applications.

Among the various strategies utilized for temperature-dependent regulation of gene expression are RNA thermometers (RNATs), which have been shown to be useful in the development of temperature-dependent genetic circuits [6,7,8]. RNATs are cis-encoded regulatory elements that modulate translational efficiency in response to environmental temperatures. This modulation is achieved through a mechanism in which Shine–Dalgarno sequences are incorporated into inhibitory structures, physically hindering ribosome binding and repressing translation at lower temperatures. As temperatures increase, these structures within RNATs are destabilized, progressively relieving the repression of translation initiation. Interestingly, the activity of RNATs is not modulated by the presence or absence of a ligand [1,2]. The ability of an RNAT to regulate expression is solely dependent on its chemical structure, which determines the differential stability of a specific inhibitory structure at different environmental temperatures.

From a synthetic biology standpoint, insights from temperature-sensing mechanisms in nature have provided a foundation for designing conditional gene expression tools [3,4,5]. In this context, there is growing interest in developing and applying temperature-controlled recombinases capable of performing irreversible DNA modifications in response to external temperature changes [6,7,8]. These recombinases can be engineered to recognize specific DNA sequences and induce targeted genetic alterations, such as insertions, deletions, or inversions, upon exposure to defined temperature thresholds. This approach enables precise modulation of gene expression through recombinase-mediated events, harnessing the inherent advantages of temperature-based regulation.

In contrast to light-inducible systems, particularly those relying on shorter wavelengths, which exhibit limited penetration into deep tissues and may result in uneven activation confined to superficial regions, thermally inducible recombinases provide distinct advantages. Heat can be uniformly applied to whole organisms or cell cultures, enabling homogeneous activation of the recombinase across all target cells, which may be particularly beneficial in specific experimental contexts. Moreover, thermal induction within an appropriate temperature range can avoid the potential harmful effects of intense or prolonged light exposure on cells, thereby reducing the risk of phototoxicity.

However, it is important to note that thermal induction also presents challenges, such as the need for precise temperature control to prevent adverse effects on cells or tissues, as well as the proper design of genetic circuits required to achieve accurate control of recombinase expression in response to temperature changes.

The present study was conducted to characterize the control of recombination events in response to different temperature conditions in Escherichia coli. This characterization focuses on the analysis of key aspects of the genetic architecture of such circuits, both for applications in liquid cell cultures and on 2D surfaces, by enabling the creation of precise thermo-spatial patterns.

Although several possible recombinases have been identified [9,10,11,12,13,14], the present study built thermo-dependent genetic circuits using the mycobacteriophage serine recombinase Bxb1, which catalyzes site-specific recombination between its corresponding *attP* and *attB* recognition sites. Depending on the relative orientation of the *attP* and *attB* sites, the reaction can result in excision, inversion, or integration of DNA sequences between these sites [15,16,17]. Specifically, excision events were analyzed as a representative case study in this work.

To investigate the performance of gene expression mediated by recombinases expressed in response to temperature changes, a library of genetic circuits was constructed, each expressing a gene of interest (GOI) regulated by the Bxb1 recombinase. The expression of Bxb1 is, in turn, temperature-dependent due to the presence of the FourU RNA thermometer sequence, derived from *Salmonella enterica* serovar Typhimurium [18,19,20,21], located upstream of the Bxb1 coding sequence.

## 2. Results

The minimal genetic architecture of a thermo-dependent genetic expression system includes a promoter, whether constitutive or regulatable, followed by the RNAT sequence, and the Bxb1 coding region. One of the main challenges in constructing these types of genetic circuits is the basal expression associated with promoter leakiness. This can result in the unintended and spontaneous expression of recombinases, leading to recombination events occurring independently of external inputs, such as temperature, and undermining the intended precise control of these circuits. The impact of recombinations arising from the basal (i.e., unregulated) expression of recombinases on the behavior of various genetic circuits has been discussed in multiple publications [22,23,24].

Consequently, optimizing genetic architecture is essential, with particular attention to minimizing basal expression by selecting an appropriate promoter activity upstream of the FourU sequence, thereby ensuring that gene expression remains tightly regulated and is activated only under the desired thermal conditions.

### 2.1. Effects of Basal Promoter Activity on Expression of Thermo-Regulated Genes

The effects of promoter activity on unintended recombination events were assessed by constructing a set of genetic circuits. Initially, a very simple circuit C1 was built in *E. coli* to characterize the effect of promoter activity on FourU performance (see Figure S1a). This circuit contains a high-copy-number plasmid that expresses GFP as a reporter downstream of a FourU sequence. A rhamnose-inducible promoter is positioned upstream of the FourU sequence. In these circuits, GFP expression is independent of Bxb1-mediated recombination, allowing for a straightforward evaluation of the promoter’s effect on the temperature-dependent expression of GFP (see Supplementary Materials for genetic details).

The performance of the FourU sequence was initially analyzed under two extreme conditions: in the absence of rhamnose and with 2% rhamnose, the latter allowing maximal promoter activity. Figure S1b,c present the GFP levels at various temperatures in circuit C1 under these conditions. At 0% rhamnose, circuit C1 demonstrated no evident temperature-dependent relationship with GFP levels (Figure S1b), attributed to the minimal transcriptional activity of the PrhaB promoter. Conversely, at 2% rhamnose, GFP expression exhibited a clear temperature-dependent pattern (Figure S1c). Notably, GFP expression was negligible at temperatures below 30 °C but increased at higher temperatures (see Materials and Methods for experimental details).

Based on these findings and consistent with prior results [25,26], a temperature of 20 °C can be considered the OFF state for FourU activity, while temperatures at or above 37 °C correspond to the ON state. Furthermore, these results underscore the importance of promoter activity in modulating the relationship between temperature and gene expression.

The sensitivity to temperature increased with enhanced promoter activity (Figure S1d). Elevating rhamnose concentrations, which augment promoter activity [27], led circuit C1 to exhibit a more pronounced response to temperature variations. This enhancement resulted in a greater differentiation in GFP levels between the OFF state at 20 °C and the ON state at 37 °C, culminating in a 5.4-fold increase in gene expression. However, heightened promoter activity was also correlated with increased basal protein expression in the OFF state (20 °C), with basal expression being 2.5-fold higher in the circuit at maximum promoter activity (>0.25% rhamnose) compared to minimal promoter activity (0% rhamnose).

### 2.2. Effects of Promoter Activity and Cellular Growth Phase on the Expression of Genes Modulated by Thermo-Regulated Recombinases

**Figure 1:**
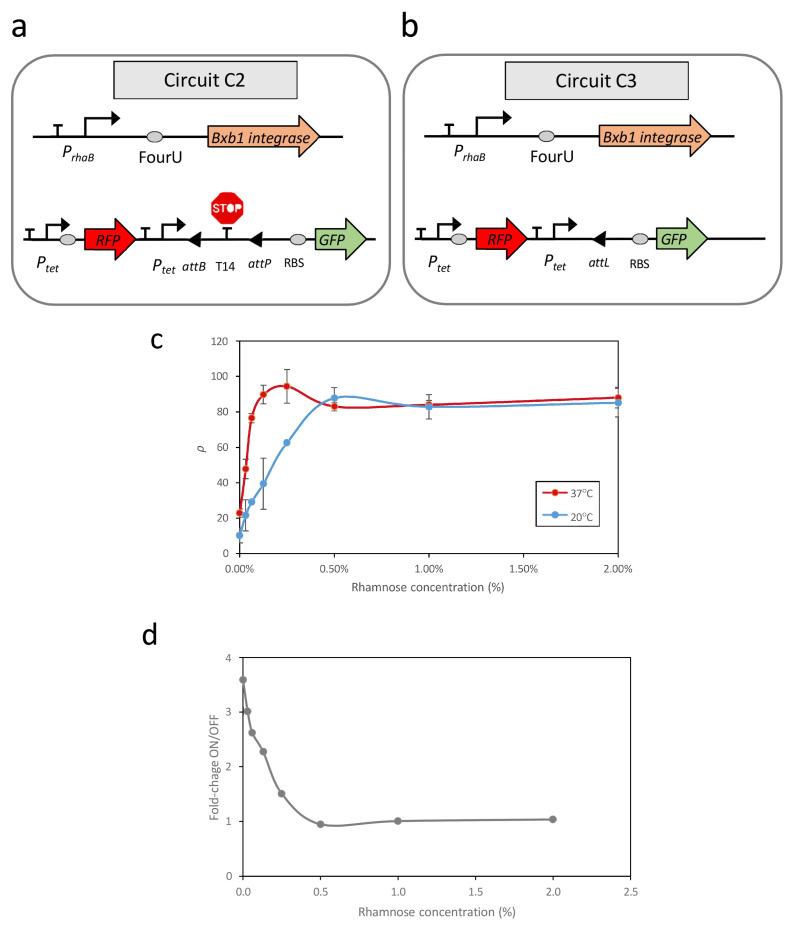
Schematic representation of the genetic circuits used in this study (**a**,**b**). (**c**) Recombination efficiencies at high (37 °C) and low (20 °C) temperatures in response to increasing induction of the rhamnose-dependent promoter. (**d**) Fold changes between the OFF and ON states at different rhamnose concentrations. Data represent the mean of three independent experiments; error bars indicate the standard deviations of these experiments.

To assess the relationship between promoter activity and recombination events across various temperatures, two additional genetic circuits, C2 and C3, were constructed in *E. coli* (see Figure 1).

Circuit C2 (Figure 1a) consisted of two modules housed within distinct plasmids. The first module, contained in a high-copy-number plasmid, functioned as a reporter circuit for excision events. This excision reporter circuit comprised a constitutive Ptet promoter positioned upstream of a double terminator sequence (T14) flanked by the attP and attB recognition sites. Downstream of this arrangement, a ribosomal binding site (RBS) sequence and a GFP gene were inserted as a reporter. The presence of T14 between the promoter and RBS prevented GFP expression. However, upon expression of the Bxb1 recombinase, the T14 sequence was excised, leading to GFP expression. Additionally, this plasmid constitutively expressed a Red Fluorescent Protein (RFP) under the control of the Ptet promoter, independent of temperature.

The second module, housed in a low-copy-number plasmid, was responsible for recombinase expression. Specifically, this module comprised the PrhaB promoter, followed by the FourU sequence and the Bxb1 coding region. Consequently, the transcription level of the Bxb1 coding region at each temperature can be modulated based on the concentration of rhamnose present in the medium.

Utilizing GFP as a reporter to assess recombination efficiency offers significant advantages, particularly by enabling straightforward in vivo monitoring of recombination events [28,29]. However, it is essential to consider that GFP fluorescence can be influenced by factors not directly related to the genetic architecture of the circuits. Specifically, GFP fluorescence levels are inherently sensitive to temperature variations [30,31] independently of regulatory mechanisms such as the FourU RNA thermometer. This intrinsic thermosensitivity must be considered when using GFP to quantify recombination efficiency, as temperature fluctuations may affect fluorescence intensity and potentially lead to inaccurate interpretations. As a result, directly comparing GFP levels measured at different temperatures to assess recombination efficiency is not reliable.

In addition to temperature, other factors may also influence GFP levels independently of recombination [32,33]. It is important to consider whether the excision of the plasmid fragment containing the T14 terminator sequence could alter the structure of the plasmid, potentially affecting its replication rate and, consequently, the levels of GFP detected.

To address these limitations, circuit C3 (Figure 1b) was constructed. This circuit contains the same Bxb1 expression module as circuit C2; however, in the module responsible for reporting excision events, the terminator sequence T14 flanked by attB and attP sites was replaced with an attL site. The attL site is the product of a recombination event between attP and attB mediated by Bxb1. Therefore, circuit C3 represents a condition equivalent to 100% recombination of the attP-attB sites in circuit C2 and serves as a reference for maximum recombination efficiency.

First, to evaluate whether the plasmid population is affected by recombination events, RFP levels were measured at different temperatures in circuits C2 and C3. In both circuits, RFP expression is not directly regulated by Bxb1; therefore, significant changes in fluorescence levels as a function of temperature can be associated with alterations in plasmid copy number. Figure S2 shows the RFP levels measured at 20 °C and 37 °C in both circuits.

Results show that although RFP fluorescence is temperature-dependent [30], no statistically significant differences were observed between the fluorescence levels of circuits C2 and C3 at either temperature. Welch’s *t*-test yielded *p*-values of 0.259 at 20 °C and 0.329 at 37 °C. Given that circuit C3 does not undergo plasmid modification through recombination, these findings suggest that recombination events do not introduce significant changes in plasmid population or stability.

The recombination efficiency of the strain carrying circuit C2 at low (20 °C) and high (37 °C) temperatures across a range of rhamnose concentrations can be quantified according to the following equation (see Section 4 for details):

(1) $$\rho =100\cdot \frac{F(T,I)}{F_{max}(T,I)}$$

Evaluation of *ρ* revealed a strong correlation between promoter activity, dependent on rhamnose concentration, and the recombination efficiency of excision events (Figure 1c). At 37 °C, with FourU in the ON state, elevated levels of promoter transcription corresponded to increases in GFP levels until saturation was achieved. A similar qualitative relationship, however, was observed at 20 °C, with higher levels of promoter induction corresponding to an increased number of excision events until saturation was achieved. Despite the system being expected to remain in the OFF state at 20 °C, with few recombination events, the rate of unwanted recombinations in this state increased rapidly as promoter activity increased. Thus, at rhamnose concentrations above 0.5%, there was no separation between OFF and ON states. These findings indicated that the design of genetic circuits based on recombination events in a temperature-dependent manner can result in unintended recombination events at lower temperatures, depending on the promoter activity upstream of the RNAT sequence. Evaluation of the relationship of fold-changes between the OFF and ON states, corresponding to 20 °C and 37 °C, respectively, and the level of promoter induction showed that the maximum difference between ON and OFF states occurred at minimal promoter activity, specifically 0% rhamnose (Figure 1d). This result was unexpected because, in circuit C1, there was no clear response to temperature in the OFF state (Figure S1b).

**Figure 2:**
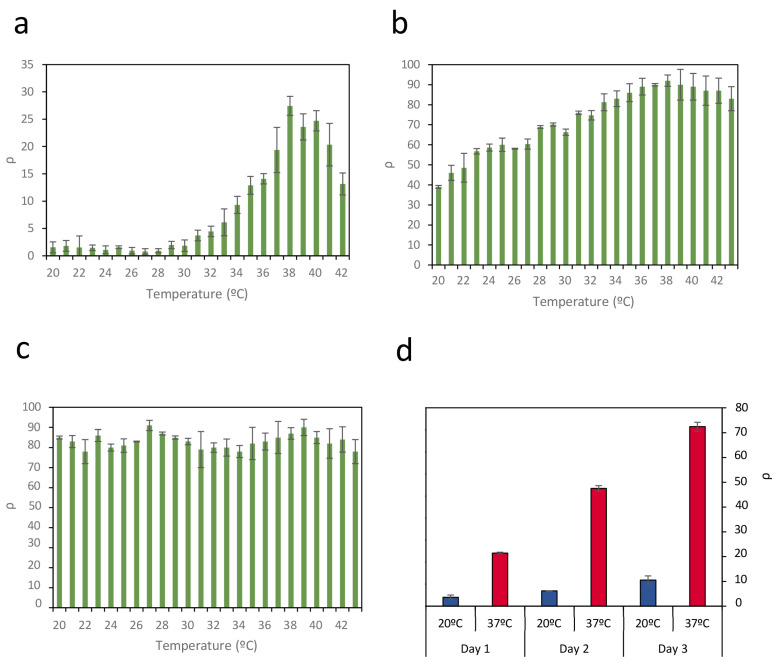
Efficiency of recombination *ρ* in circuit C2 across a wide temperature range under three levels of promoter activity: (**a**) 0% rhamnose, (**b**) 0.13% rhamnose, and (**c**) 2% rhamnose. Thermal sensitivity is strongly influenced by promoter activity. Data represent the mean of three independent experiments; error bars indicate the standard deviation of these experiments. (**d**) Evolution of recombination efficiency *ρ* at 37 °C (red bars) and at 20 °C (blue bars) during three successive 24-h cultures, where the initial inoculum of each culture comes from the previous culture once it has reached a steady state. Data represent the average of three independent experiments. Error bars indicate the standard deviation of three independent experiments.

For illustrative purposes, Figure 2 shows representative data of the recombination efficiency (*ρ*) in circuit C2 across a wide temperature range under three levels of promoter activity: 0% rhamnose (Figure 2a), 0.13% rhamnose (Figure 2b), and 2% rhamnose (Figure 2c). The results indicate that thermal sensitivity is strongly influenced by promoter activity. At low promoter activity (0% rhamnose), recombination is minimal below 30 °C and reaches a maximum efficiency of *ρ* = 27% at higher temperatures. Although this configuration provides the most favorable performance in terms of clear separation between low and high-temperature responses, along with minimal protein expression in the OFF state, the maximum recombination efficiency remains relatively low.

In contrast, at intermediate promoter activity (0.13% rhamnose), a significant increase in recombination events is observed, with a maximum efficiency of *ρ* = 90%. However, increased promoter activity also leads to substantial recombination at lower temperatures, thereby reducing the separation between the OFF and ON states. When promoter activity is very high (2% rhamnose), the circuit exhibits a complete loss of temperature sensitivity.

It is important to highlight that, although both circuit C1 and the PrhaB–FourU–Bxb1 module in circuit C2 respond similarly to PrhaB promoter activity, consistent with previous results [34], their behavior diverges in terms of dynamic response. In circuit C2, increasing PrhaB activity reduces the OFF–ON separation of the circuit, as shown in Figure 1d, which translates into a loss of sensitivity of circuit C2’s response to temperature changes when PrhaB promoter activity is elevated. Consequently, the activity of the promoter upstream of the FourU–Bxb1 module appears to be a crucial parameter determining the circuit’s thermal response.

However, although the greatest separation between OFF and ON states is achieved in circuit C2 with 0% rhamnose, the maximum recombination efficiency *ρ* under these conditions reaches only 27%. Although the reason for not achieving 100% of the possible recombinations is not clear, a relationship between growth phase and the increase in GFP levels due to recombination events was observed. The entry of bacterial cultures into the stationary phase affects the expression of genes modulated by recombination events and the maintenance of GFP levels (Figure S3). This finding is consistent with previous results indicating that entering the stationary phase can be accompanied by significant reductions in plasmatic protein synthesis [34,35,36], affecting both Bxb1 and GFP.

To determine if the growth phase could be a limiting factor in recombination events, *E. coli* containing circuit C2 was cultured in medium containing 0% rhamnose at 20 °C and 37 °C. Once each culture reached the stationary phase, an aliquot was inoculated into fresh medium; this process was repeated for 3 days. The same procedure was concurrently applied to circuit C3. The recombination efficiency *ρ* was calculated each time the culture entered the stationary phase (Figure 2d). At 20 °C, corresponding to the OFF state, there was a slight increase in recombination rate, possibly due to the low basal expression of Bxb1. At 37 °C, however, recombination rates of 23% were achieved after the first day, similar to the rates shown in Figure 2a. However, as the growth was repeated on successive days, recombination levels reached 73%. These results suggest that recombination events accumulate each time the culture enters the growth phase until it again reaches the stationary phase. This outcome suggests that the duration of the exponential growth phase in a culture can be a limiting factor for inducing recombination events in response to temperature changes.

The results presented above indicate that, in liquid cultures, several key parameters govern the precise control of recombination events in temperature-inducible genetic circuits. Specifically, promoter activity, the duration of temperature pulses to which the circuit is exposed, and the length of the exponential growth phase are three critical factors that must be carefully considered when implementing genetic circuits designed to induce irreversible DNA modifications in response to thermal cues.

Moreover, although genetic architectures based on low promoter activity (e.g., PRhaB at 0% rhamnose) only achieve partial recombination across available sites, their lack of basal activity at low temperatures and greater dynamic range between OFF and ON states make them well-suited for constructing complex genetic devices requiring clear binary behavior.

### 2.3. Recombination upon Different Temperature Pulses

**Figure 3:**
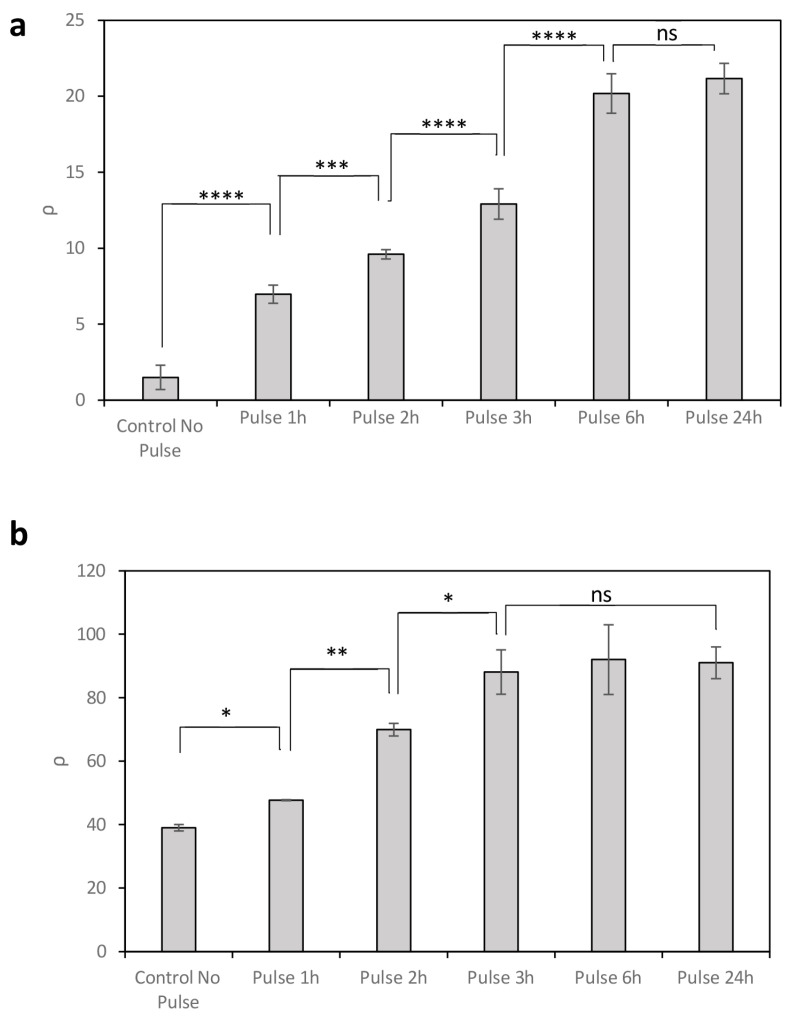
Recombination efficiency *ρ* resulting from the expression of Bxb1 under temperature pulses of varying duration at 37 °C, with 0% rhamnose (**a**) and 0.13% rhamnose (**b**). Data represent the mean of three independent experiments; error bars indicate the standard deviation of these experiments. * *p* < 0.05, ** *p* < 0.01, *** *p* < 0.001, **** *p* < 0.0001; ns, not statistically significant.

Levels of Bxb1 expression in circuit C2 have been shown to depend on promoter activity and can be modulated by temperature. However, Bxb1 concentrations are influenced not only by temperature but also by the duration of the thermal pulse. To investigate the effect of pulse duration on recombination efficiency, circuits C2 and C3 were exposed to 37 °C temperature pulses of varying lengths, following initial cultivation at 20 °C under conditions of low promoter activity (0% rhamnose) and higher promoter activity (0.13% rhamnose), respectively (Figure 3).

In circuit C2 with 0% rhamnose, longer temperature pulses led to increased recombination efficiency (*ρ*) due to the accumulation of intracellular Bxb1 (Figure 3a). Maximum *ρ* levels were reached after 6 h, with no significant increase observed for longer exposures. In contrast, under conditions of higher promoter activity (0.13% rhamnose), although high GFP expression was already detected at 20 °C (no pulse), further increases were observed following temperature pulses at 37 °C (Figure 3b). In this case, maximum recombination efficiency was achieved with shorter pulses, reaching saturation after only 3 h. This rapid response is likely due to the high number of recombination events occurring prior to pulse application, consistent with the elevated translation rate conferred by the strong upstream promoter.

These results show that, beyond promoter activity and temperature, the duration of thermal induction provides an additional level of control over recombination dynamics.

### 2.4. Spatial Control of Temperature-Dependent Recombination Events

The use of paper-based surfaces to develop bacteria-based biosensors enables the creation of low-cost, portable, and disposable devices, ideal for applications such as environmental monitoring [37], contaminant surveillance [38], or point-of-care diagnostics [39], among others. Moreover, these architectures benefit from division of labor among different cell types [40], thereby reducing the genetic complexity required per cell. The integration of genetically encoded, temperature-controlled systems represents a promising advance in this field. Specifically, an important advantage of temperature- inducible recombinases is their capacity to drive spatially resolved recombination, enabling the generation of temperature-dependent spatial patterns, which could introduce new functionalities to these types of devices.

To investigate this, the effect of temperature on surface-based recombination events was evaluated. Building on prior results, spatial control of temperature-dependent recombination was assessed using circuit C2 in the absence of rhamnose (0%), with circuit C3 serving as a reference for quantifying the recombination efficiency.

Initially, recombination efficiency was quantified on two paper surfaces: one uniformly inoculated with an *E. coli* strain harboring circuit C2, and the other with circuit C3. Both were incubated at 20 °C. Subsequently, the relationship between temperature and GFP on a surface culture was assessed by exposing these surfaces to different temperatures. GFP levels following exposure to different temperatures were measured by scanning the paper surface containing the cellular circuits with an HTX BioTech Synergy plate reader. Since direct measurement of OD is not feasible in surface-based paper cultures, cell concentration was estimated based on the levels of constitutively expressed RFP from circuits C2 and C3. In this way, surface recombination efficiency *ρS* can be calculated as follows (see Materials and Methods for details):

(2) $$\rho_{S}=100\cdot \frac{F^{S}(T,I)}{F_{max}^{S}(T,I)}$$

**Figure 4:**
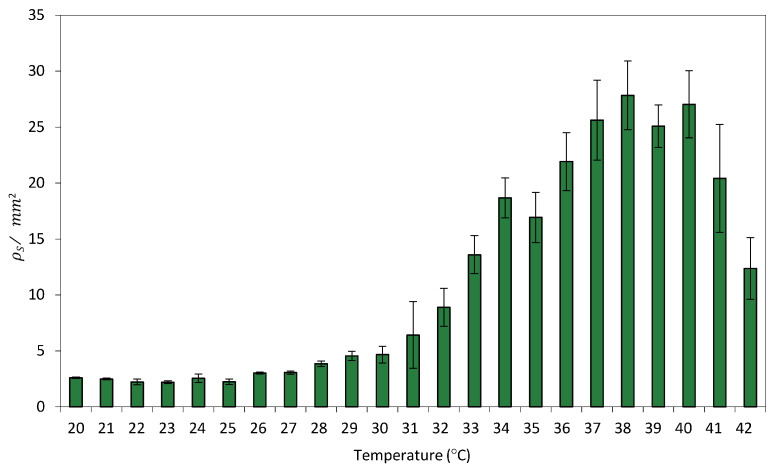
Relationship between GFP/mm^2^ and temperature in Circuit C3 uniformly distributed on a paper surface. Data represent the mean of three independent experiments. Error bars indicate the standard deviation of the three independent experiments.

Because circuit C2 exhibits a greater separation between OFF and ON state fluorescence levels at 0% rhamnose, all surface-based experiments were conducted under this condition. Figure 4 shows the relationship between the average *ρ*_*S*_ per unit area, i.e., *ρ_S_*/mm^2^ and temperature.

Despite the fact that bacterial growth on solid surfaces presents several limitations compared to agitated liquid cultures [41], limitations that can influence cellular physiology and gene expression, particularly in synthetic circuits sensitive to environmental cues, a comparison of this transfer function with its equivalent in liquid culture (Figure 2) revealed a strong similarity in recombination efficiency between surface- and liquid-grown cells. This suggests that spatial confinement does not significantly impact the efficiency of recombination events.

**Figure 5:**
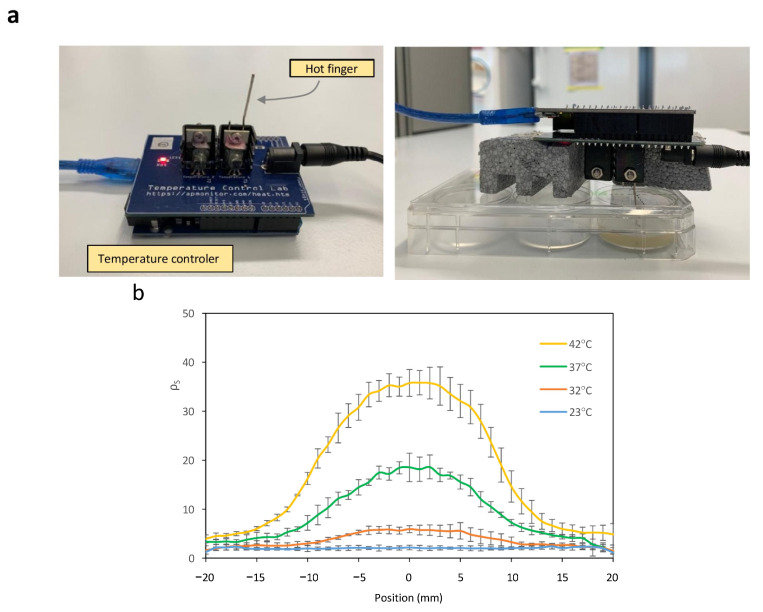

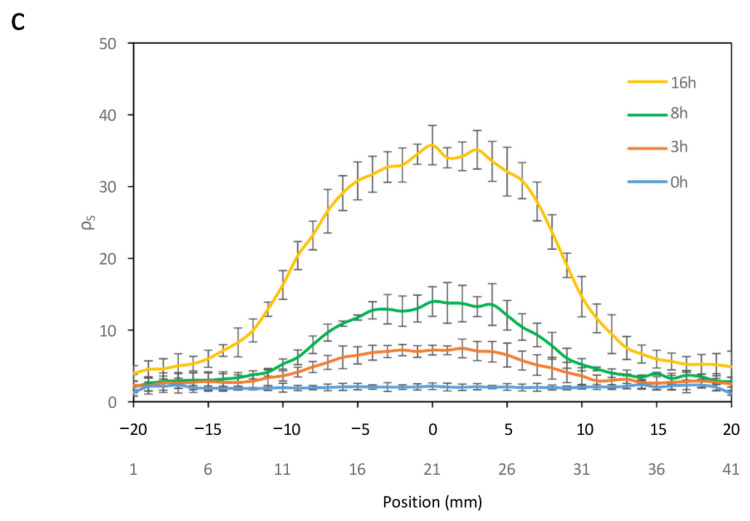
(**a**) Experimental setup of a hot finger for the induction of recombination events at a localized point on the surface. The temperature control of the hot finger is achieved using an Arduino temperature controller. (**b**) Cross-sectional view of *ρ_S_* levels at each spatial point within spots induced at different temperatures. (**c**) Cross-sectional view of *ρ_S_* levels at each spatial point within spots induced at 42 °C for varying time periods. Error bars indicate the standard deviation of the three independent experiments.

To investigate the generation of spatial patterns through temperature-dependent expression of Bxb1, *E. coli* cells harboring circuits C2 and C3 were cultivated on a paper surface at 20 °C. A 1 mm diameter hot finger maintained at 37 °C was used to create discrete thermal spots at defined locations (see Materials and Methods for experimental details for experimental setup). Figure 5a shows an image of the experimental setup. The surface recombination efficiency *ρ_S_* was quantified at each position. Owing to the radial symmetry of heat diffusion, a transverse section of the resulting spatial temperature profile was used to characterize the formation of recombination-induced spots around the hot-finger contact point. Figure 5b shows the spatial distribution resulting from the thermal gradient generated on the paper surface after hot-finger application at position (0, 0). Cells were exposed to localized temperatures of 23 °C, 32 °C, 37 °C, and 42 °C for 16 h. Localized thermal stimulation triggered recombination in cells that experienced temperatures above a threshold value. As the distance from the application site increases, the temperature decreases accordingly, leading to a reduction in recombination efficiency. Higher temperatures were associated with increased recombination levels, accompanied by a slight rise in basal activity.

Finally, the generation of spatially localized spots in response to temperature pulses of varying duration was examined. Circuits C2 and C3 were cultivated on a paper surface at 20 °C, followed by the application of a 42 °C hot finger for different time intervals. Figure 5c shows the resulting spot profiles, expressed as *ρ_S_*, as a function of pulse duration. Recombination efficiency increased with longer exposure times, although basal recombination levels also exhibited a slight rise with extended pulse durations.

These results demonstrate that spatial gene expression patterns can be generated through localized thermal activation and that such patterns can be precisely tuned by modulating both the applied temperature and the duration of exposure at each specific location on the surface.

Together, these findings demonstrate that minimal genetic circuits regulated by the expression of a temperature-responsive recombinase provide multiple layers of control, including promoter strength, temperature, and pulse duration, that enable precise modulation of gene expression. This framework is applicable to both liquid cultures, where regulation affects the entire population, and to surface-based systems, where localized inputs enable spatially differentiated control. These features make such synthetic devices promising tools for a wide range of applications in both homogeneous and spatially structured environments.

### 2.5. Thermal Configuration of Spatial Patterns

One potential application for cellular systems expressing a GOI, such as GFP, in response to temperature is the formation of spatial patterns defined by temperature differences between different points on a surface. Because this type of application requires good resolution between points on the surface at different temperatures, the cellular system should not generate a basal signal that could obscure the pattern being configured. This requirement is met by a genetic architecture based on thermo-regulated recombinases, along with a reduction in the duration of maintenance of the temperature pattern to obtain the corresponding cellular response.

To investigate the formation of spatial patterns, *E. coli* harboring Circuit C2 was cultured at 20 °C and uniformly spread onto a 27 mm long strip of paper in the absence of rhamnose.

**Figure 6:**
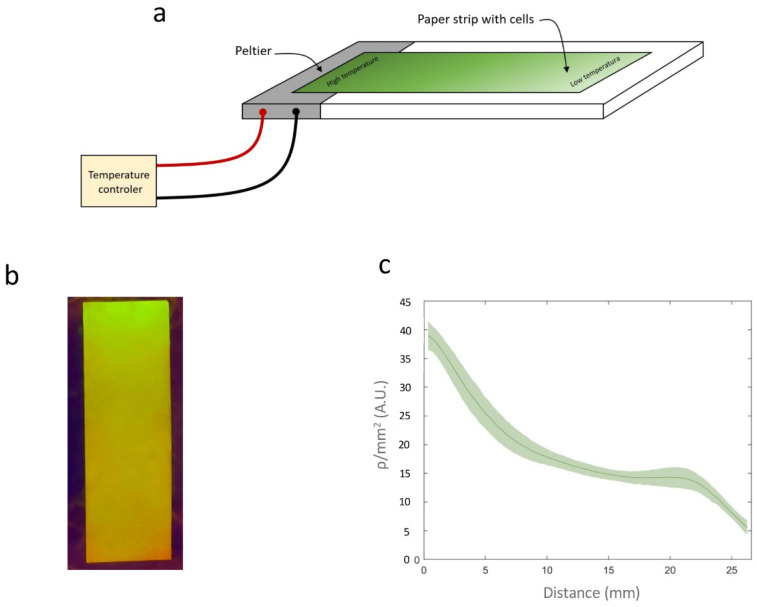

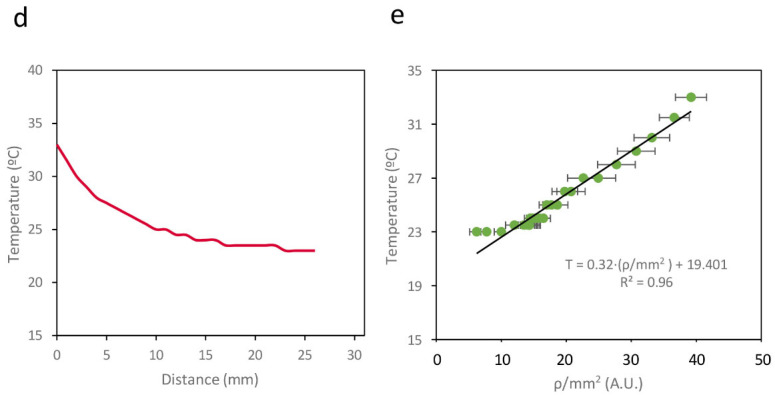
(**a**) Schematic representation of the experimental setup. (**b**) Image of a paper strip containing a cell culture from Circuit C2 subjected to a temperature gradient. (**c**) *ρ*/mm^2^ values on the paper strip. (**d**) Temperatures measured with a thermocouple along the surface of the temperature gradient. (**e**) Relationship between *ρ*/mm^2^ and temperature measured at different points on the surface. Data represent the mean of three independent experiments. Error bars indicate the standard deviation of the three independent experiments.

A similar setup was prepared for Circuit C3. A temperature gradient was generated by applying two different temperatures (42 °C and 23 °C) at two points 30 mm apart on a Petri dish containing Lysogeny Broth (LB) agar. The paper strips were placed between these two points, 4 mm from the 42 °C point. This configuration was maintained for 3 h, after which the paper strips were incubated at 20 °C for 18 h. Figure 6a shows an image of the paper strip containing Circuit C2 under a transilluminator, where a clear gradient in fluorescence intensity can be observed. The average recombination efficiency (*ρ*/mm^2^) along the strip was measured using a Synergy MX microplate reader (BioTek Instruments, Winooski, VT, USA). Figure 6b shows how *ρ*/mm^2^ varies with the distance from the high-temperature point. Direct measurement of the temperature gradient on the surface using a thermocouple connected to an Amprobe 38XRA multimeter (Amprobe, Everett, WA, USA) (Figure 6c) revealed a linear correlation between the measured temperature and *ρ*/mm^2^ on the paper strip, with R^2^ = 0.96 (Figure 6d). This linear relationship enables a direct conversion between *ρ*/mm^2^ values and temperature, demonstrating that this device can be used to record temperature gradients on paper surfaces.

### 2.6. Temperature-Controlled Cellular Release Systems

One potential application of these circuits, based on the temperature dependence of recombinase-mediated gene expression, is the creation of temperature-controlled cellular release systems.

Specific microorganisms can be genetically engineered to express enzymes or other compounds of interest [42]. Once these compounds are produced, however, it may be necessary to release them from the intracellular environment, including through the expression of lytic genes under specific conditions [43,44]. Inducing the expression of these lytic genes can result in disruption of the cell walls, allowing the compounds of interest to be released into the surrounding medium, where they can be harvested [45].

The expression of lytic genes can be induced by altering temperature, a more cost-effective option than chemical inducers. Moreover, temperature changes can be uniformly applied throughout the entirety of a cellular population. These systems require that cellular lysis occurs above a certain temperature, with basal expression being close to 0 at low temperatures. An architecture based on low-activity promoters, such as Circuit C2, is suitable for these applications, as there are no indications of significant basal activity at low temperature (20 °C), preventing unintended cell lysis. By contrast, protein expression was at a sufficiently high temperature (37 °C), resulting in the efficient induction of cell lysis.

This hypothesis was tested by constructing two new *E. coli* strains. Specifically, Circuit C7 (Figure S4a) and Circuit C8 (Figure S4b) were designed as direct derivatives of Circuits C1 and C2, respectively, both combining the GFP sequence with a lytic gene from bacteriophage ϕX174 in a bicistronic construct [46,47]. These new cell strains were cultured in liquid medium at 20 °C until reaching an OD_660_ of 0.9. Subsequently, the temperature of both cultures was raised to 37 °C for 3 h. Finally, the cultures were maintained at 20 °C for several days. All experiments were conducted at 0% rhamnose to ensure minimal activity of the PrhaB promoter, thus preventing unintended recombination at 20 °C and avoiding cell lysis at this low temperature. Use of higher rhamnose concentrations led to recombination at 20 °C, resulting in undesired expression of the lysis gene, which compromises cell growth at low temperature.

This study hypothesized that *E. coli* bearing Circuit C7 would express very low levels of the lytic protein, in agreement with the results in Figure S1b. By contrast, the expression of Bxb1 by *E. coli* bearing Circuit C8 was expected to enable significant expression of the lytic gene, consistent with the results in Figure 2a. Expression of the lytic gene would therefore result in cell wall rupture and the release of intracellular content into the external environment. Consequently, cell lysis should result in increased levels of extracellular GFP and decreased levels of intracellular GFP. The efficiency of temperature-induced cellular lysis could therefore be monitored by measuring extracellular GFP levels (see Materials and Methods for experimental details).

**Figure 7:**
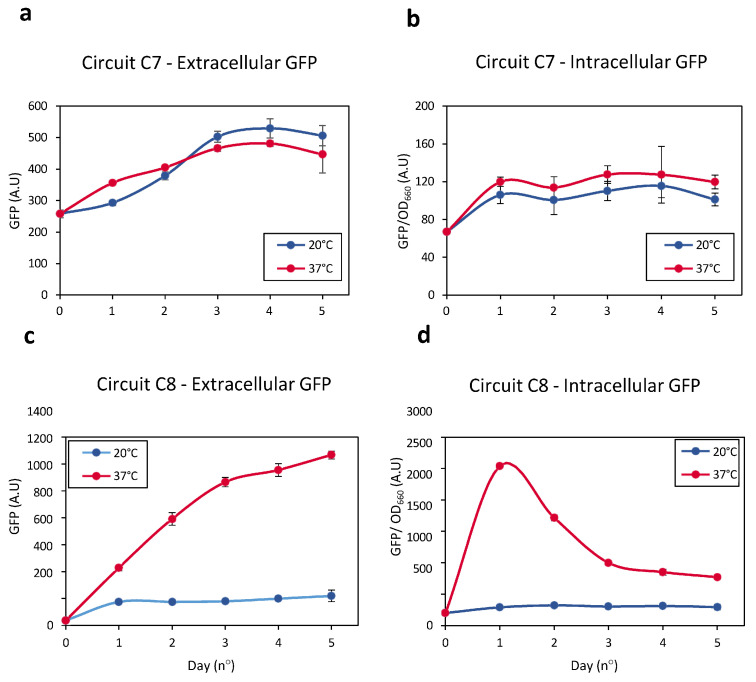
(**a**) Levels of extracellular GFP released by Circuit C7 at 20 °C (blue line) and 37 °C (red line) resulting from cellular lysis. (**b**) Intracellular GFP levels measured in Circuit C8 at 20 °C (blue line) and 37 °C (red line) as a result of cellular lysis. (**c**) Levels of extracellular GFP generated by Circuit C8 at 20 °C (blue line) and 37 °C (red line) resulting from cellular lysis. (**d**) Intracellular GFP levels released by Circuit C8 at 20 °C (blue line) and 37 °C (red line) as a result of cellular lysis. Data represent the mean of three independent experiments. Error bars indicate the standard deviation of the three independent experiments.

Changes in extracellular and intracellular GFP levels were therefore monitored in *E. coli* bearing Circuits C7 and C8 (Figure 7). GFP levels in the extracellular medium of Circuit C7-bearing bacteria at 37 °C did not differ significantly from the levels observed at 20 °C (Figure 7a,b). Similarly, intracellular GFP levels were similar at both temperatures. Taken together, these results indicate that the lytic gene was not significantly induced when it is located directly downstream of a low-activity promoter and the FourU sequence. These results were consistent with those observed in Figure S1b.

Increasing the temperature of *E. coli* bearing Circuit C8 from 20 °C to 37 °C significantly enhanced the expression of the lytic gene over time, resulting in efficient cell wall rupture and the release of intracellular contents into the external medium. The higher extracellular GFP levels at 37 °C than at 20 °C indicated that cellular lysis was greater at the higher temperature (Figure 7c). Evaluation of intracellular GFP levels showed that, during an initial phase, these levels increased over time at 37 °C, indicating that Bxb1 levels increased as the temperature increased from 20 °C to 37 °C (Figure 7d). Bxb1 expression, however, also increased the expression of the lytic gene, resulting in the release of the intracellular contents, including GFP, into the external medium. Thus, intracellular GFP levels decreased as extracellular GFP levels increased.

These results demonstrate that a system based on thermo-regulated recombinases allows for efficient induction of cellular lysis in response to a temperature increase, ensuring that basal expression at low temperatures, i.e., 20 °C, is very low and thus does not compromise cell integrity.

## 3. Discussion

Genetic circuits involving temperature-dependent recombinases can be used in multiple applications across various fields, ranging from biotechnological production of targeted molecules to advances in biomedical applications. The precise design of these genetic circuits is crucial to prevent the occurrence of unintended recombination events. The present study analyzed the key elements required to construct minimal cellular devices for temperature-dependent recombinase expression.

Three critical parameters must be considered to construct these genetic devices: (i) promoter activity, (ii) temperature, and (iii) the duration of temperature pulses. This study demonstrated the ability to establish two temperature levels under certain conditions. The first, termed the OFF state, was characterized by the absence of recombination events, whereas the second, termed the ON state, included active recombination events.

Separation between the OFF and ON states was found to depend on the activity of the promoter responsible for Bxb1 recombinase expression, with high promoter activity reducing the separation between these two states. Specifically, the promoter with the lowest activity was the most effective in creating temperature-dependent ON/OFF systems.

Recombinases have an amplifying effect on the temperature-dependent response of these types of devices. Although basal expression can be minimized by using promoters with low activity, their low activity renders genetic circuits virtually insensitive to temperature changes. The introduction of an intermediate element, such as a recombinase expressed in response to temperature, was found to significantly enhance the response of these genetic circuits to temperature variations while maintaining minimal basal activity.

Temperature regulation of recombinase expression holds multiple advantages due to its cost-effectiveness, ease of control, and applicability. Temperature-dependent recombinases can induce gene expression in a bulk cell culture setting, such as in a bioreactor, requiring a change in temperature. 

Alternatively, the use of this system can be precisely applied to more complex configurations, such as when selective recombinations are generated in designated parts of the cellular device by applying localized temperature patterns at specific points on the surface.

This study explored some illustrative examples: the formation of spatial patterns generated by temperature patterns on the surface, the creation of a paper-based device capable of recording the temperature gradients on a surface, and the development of a genetic device capable of inducing cellular lysis in response to a temperature increase.

In all of these examples, the genetic architecture used demonstrated good performance.

Additional studies are required, however, to design and implement more complex devices in order to determine their potential and limitations. A highly promising type of surface-based application is the development of reprogrammable cellular devices with distributed computing capabilities on 2D surfaces [40]. In such implementations, reprogrammable systems could be created through the application of surface temperature patterns, inducing DNA modifications in a subset of cells. This approach could also potentially facilitate the creation of sensors capable of detecting temperature changes and triggering distinct cellular responses based on these temperatures.

Despite this being an initial exploration of the possibilities of genetic circuits based on the thermo-dependent expression of recombinases, the outcomes of this study underscore the potential for developing such cellular devices. Nevertheless, these results are limited to the study of excision events with a specific type of recombinase, namely Bxb1, in *E. coli*. More comprehensive understanding and broader application of these systems requires evaluations of other types of recombination events, such as DNA inversion, other recombinases, and other cell types. Studies are also needed to analyze the relationship between recombinase expression and their ability to modify DNA at each phase of cell culture growth, especially steady state growth, which may be a limiting factor in the use of such systems.

## 4. Materials and Methods

### 4.1. Strains, Media, and Growth Conditions

*E. coli* Top10 (Invitrogen, Waltham, MA, USA) was used in all cloning and expression experiments.

Unless stated otherwise, cells were grown in LB at 37 °C and selected with the appropriate antibiotic (chloramphenicol 35 µg/mL or kanamycin 35 µg/mL; Sigma, St. Louis, MO, USA). Bacterial strains were preserved in LB glycerol 20% (*v*/*v*) at −80 °C.

Surface experiments were performed in Petri dishes (ddbiolab) containing 20 mL LB broth with 1.2% (*w*/*v*) agar (Sigma-Aldrich, St. Louis, MO, USA), supplemented with the appropriate antibiotic. In the experiments involving paper strips, cells were uniformly spread on these strips, which were later placed on an LB agar surface.

Single colonies obtained from streaked glycerol stocks were inoculated into fresh LB medium with antibiotics and grown overnight at the required temperature with shaking (200 rpm). To correct for slower growth at lower temperatures, all measurements were performed when the culture reached an OD_660_ = 0.9.

Induction medium consisted of LB supplemented with the appropriate antibiotic and L- Rhamnose monohydrate (Sigma, USA). In the temperature pulse experiments, cells were grown from glycerol stocks at 20 °C in LB medium with the required antibiotics until they reached the stationary phase (OD_660_ = 0.9). The cells were diluted 1:10 in fresh LB medium, kept at 20 °C for 30 min, and cultured at 37 °C for 0, 1, 2, 3, or 6 h or overnight. The cells were grown overnight at 20 °C, with measurements performed the following day.

In the recombination experiments, glycerol stocks were diluted in 1 mL LB medium and cultured at 20 °C, 30 °C, or 37 °C until they reached saturation (OD_660_ = 0.9). GFP fluorescence was measured as described below. A 1 µL aliquot of each saturated culture was inoculated into 1 mL of fresh LB medium and cultured at the same temperature.

### 4.2. Building of the Genetic Circuits

All the genetic circuits were constructed using the Biobrick assembly method (Ginkgo Bioworks, Boston, MA, USA) [48] by Integrated DNA Technologies Company (San Diego, CA, USA). The constructs were built on one of the following Biobricks backbones: pSB1AK3 (high-copy plasmid with ampicillin and kanamycin resistance), pSB1AC3 (high-copy plasmid with ampicillin and chloramphenicol resistance), and pSB3K3 (low-medium copy plasmid with kanamycin resistance). All transformations were performed using a chemical method, with the sequence of genetic constructs confirmed by Sanger sequencing.

### 4.3. Fluorescence Quantification in Liquid Cultures

GFP fluorescence was measured at an excitation wavelength of 485 ± 20 nm and an emission wavelength of 528 ± 20 nm using a Synergy MX microplate reader (BioTek Instruments, Winooski, VT, USA). Sample (S) absorbance (OD660 (S)) and fluorescence (f(S)), respectively) were corrected using respective signal background (B) controls for absorbance (OD660(B)) and fluorescence (f(B)). Data are the averages obtained from three independent experiments. Reporter protein F was calculated using the following equation:

$$F=\frac{f(S)-f(B)}{{OD}_{660}\left(S\right)-OD_{660}(B)}$$

### 4.4. Fluorescence Quantification on Paper at Different Temperatures

To assess the relationships between temperature and fluorescence on the paper surface, 1 × 1 cm squared paper pieces were placed on Petri dishes containing LB agar with the appropriate antibiotics. A 200 µL aliquot of previously cultured cells grown at 20 °C until OD_660_ ≈ 0.6 was spread across the plate, resulting in a concentration of 3.44 µL/cm^2^. The plates were incubated overnight (18 h) at their specified temperatures.

Each paper square was subjected to surface scan measurement and analysis (see 2D-Surface experiments). For each individual replicate, the mean GFP value of the whole surface was measured and expressed in GFP/mm^2^ units.

### 4.5. Arduino-Based Local Heating Device

The local spot heating device consisted of a Temperature Control Lab device from Apmonitor (http://www.apmonitor.com/pdc/index.php/Main/ArduinoTemperatureControl, accessed on 4 December 2025), with some modifications. A 4.5 cm long stainless-steel rod of 1 mm diameter was inserted between the heater and the shield covering it, followed by tight binding using the built-in screw. To assess the relationship between the temperature measured by the built-in sensor and the actual temperature at the furthest end of the rod, a posterior calibration was performed using a thermocouple connected to an Amprobe 38XRA multimeter.

The Arduino code used to control the temperature in this project can be found at the following GitHub link: https://github.com/marcgonzacolell/Arduino-Temperature/blob/main/Arduino_temperature_code.ino, accessed on 4 December 2025.

### 4.6. 2D Surface Experiments

The 2D surface spot experiments were conducted in 6-Well Nunc plates (Thermo Fisher, Waltham, MA, USA) containing 4 mL of LB agar with the required antibiotics. A 2.5 cm square piece of 75 gsm White Paper (Pack of 2500) 59908 was placed on top of the LB agar.

Bacteria were cultured in LB medium containing the appropriate antibiotics at 23 °C for 6 h. A 100 µL aliquot of each cell culture was plated on each well, resulting in a concentration of 10.42 µL/cm^2^. The rod of an Arduino heater was placed upside down, penetrating through a hole on the plate, with its furthermost end in close contact with the center of the paper square (see Figure 5a).

The results of all 2D surface experiments were measured using a Synergy MX microplate reader (BioTek Instruments, USA) and a surface scan protocol and analyzed with Microsoft Excel. GFP fluorescence measurements were performed at an excitation wavelength of 485 ± 20 nm and an emission wavelength of 528 ± 20 nm. Each paper square consisted of an array of 51 × 51 points, separated by 500 µm each. The highest value of each individual array was selected, and the values of its row and column were plotted after subtracting background measurements of paper alone on LB agar.

### 4.7. Gradient Experiments

A rectangular 1 × 3 cm strip of paper was placed on top of an LB agar plate supplemented with kanamycin and chloramphenicol. A 200 µL aliquot of cells grown at 20 °C to OD_660_ ≈ 0.6 was spread homogeneously across the plate, yielding a concentration of 3.44 µL/cm^2^. Heat was applied by aPeltier cell at 4V inserted into the LB agar 4 mm from the paper stripe. Temperature diffusion across the paper was calibrated using a thermocouple connected to an Amprobe 38XRA multimeter.

### 4.8. Quantification of Recombination Efficiencies in Experiments in Liquid Culture and on Surface

In liquid experiments, assuming that recombination does not substantially alter the plasmid population, and based on the results shown in Figure S2, the recombination efficiency of circuit C2 at a given temperature can be quantified by comparing its GFP levels with those of circuit C3 under identical cultivation and temperature conditions. At a specific temperature *T*, the recombination efficiency (*ρ*) can be calculated as follows:

$$\rho =100\cdot \frac{F(T,I)}{F_{max}(T,I)}$$

Here, *F*(*T*, *I*) denotes the fluorescence level of circuit C2 at a given temperature *T* and rhamnose concentration *I*, normalized by optical density (OD); *F_max_*(*T*, *I*) represents the fluorescence of circuit C3 under the same temperature and rhamnose conditions, also normalized by OD.

Likewise, the recombination efficiency in on-surface experiments, *ρ_S_*, is given by the following expression:

$$\rho_{S}=100\cdot \frac{F^{S}(T,I)}{F_{max}^{S}(T,I)}$$

Because in surface-based experiments the OD cannot be determined to normalize GFP levels, we used the constant RFP expression per cell as the normalization factor. In this way, *F*^*S*^(*T*, *I*) represents the GFP/RFP ratio measured in circuit C2 grown on a paper surface at a given temperature *T* and rhamnose concentration *I*. Similarly, $F_{max}^{S}$(*T*, *I*) denotes the GFP/RFP ratio measured in circuit C3 under the same conditions.

### 4.9. Lytic Protein Experiments

Circuits C7 and C8 were grown from the glycerinate stocks in 2 mL LB at 20 °C, with shaking at 200 rpm, for 48 h. After reaching OD_660_ = 0.9, the cultures were diluted 1:2 by adding fresh LB medium to a total volume of 4 mL in culture tubes. Each sample was split and grown at 20 °C or 37 °C. Over the following 5 days, a 500 µL aliquot was withdrawn daily from each tube and centrifuged for 1 min at 14.000 rpm. The supernatant was decanted, and the pellet was resuspended in 500 µL of fresh LB medium.

## Data Availability

Plasmids and strains used in this study and other materials are available upon request to the authors. Correspondence and requests for materials should be addressed to J.M. (javier.macia@upf.edu).

## Supplementary Materials

The following supporting information can be downloaded at: https://www.mdpi.com/article/10.3390/ijms262412055/s1.
