# Supplementary material for: Temperature-Dependent Recombinase-Based Genetic Circuits
Source: Int J Mol Sci. 2025 Dec 15;26(24):12055. doi: 10.3390/ijms262412055 (PMC12733206; doi:10.3390/ijms262412055)
Supplement: Supplementary file 1 [file ijms-26-12055-s001.zip › ijms-3957641-supplementary.pdf]

TEMPERATURE-DEPENDENT RECOMBINASE-BASED GENETIC CIRCUITS

Gonzalez-Colell[1] M., Gomes-del Castillo M.[1], Palau-Gauthier[1] M., Macia J[1,✉]

1 Synthetic Biology for Biomedical Applications Lab, Department of Medicine and Life Sciences, Universitat Pompeu Fabra, Biomedical Research Park, Barcelona, Spain.

✉Email: javier.macia@upf.edu

1. GENETIC SEQUENCES

**Bxb1**	ATGAAACATCATCACCACCACCATCAGGCGGGCCGCGCGTTAGTGGTTATTCGTCTGAG<br>CCGTGTGACCGATGCCACCACCTCGCCGGAACGTCAGCTGGAAAGCTGTCAGCAGCTGT<br>GTGCGCAGCGCGGTTGGGATGTTGTGGGCGTAGCGGAAGACCTGGATGTGAGCGGTGCC<br>GTGGATCCGTTCGATCGCAAACGTCGCCCGAACCTGGCGCGCTGGCTGGCCTTCGAAGA<br>ACAGCCGTTTGACGTGATCGTGGCGTATCGTGTGGATCGCTTAACCCGCAGCATTCGCC<br>ATCTGCAACAGTTAGTGCACTGGGCGGAAGATCATAAAAAACTGGTGGTGAGCGCGACC<br>GAAGCGCATTTTGATACCACCACCCCGTTTGCCGCGGTGGTCATCGCCCTGATGGGCAC<br>CGTGGCGCAGATGGAATTGGAAGCAATTAAAGAACGCAATCGCTCAGCCGCGCACTTTA<br>ATATTCGTGCGGGTAAATATCGTGGCAGCCTGCCGCCGTGGGGCTACCTGCCGACTCGT<br>GTGGATGGCGAATGGCGCCTGGTGCCGGATCCCGTGCAACGTGAACGTATTCTGGAAGT<br>GTATCATCGCGTGGTGGATAACCATGAACCGCTGCATCTGGTGGCGCATGATCTGAATC<br>GCCGCGGCGTGCTGTCACCGAAAGATTACTTTGCGCAGCTGCAAGGCCGCGAACCGCAG<br>GGCCGCGAATGGAGCGCAACCGCTCTGAAACGTAGCATGATTAGCGAAGCCATGCTGGG<br>CTACGCCACCCTGAACGGCAAAACCGTGCGTGATGATGATGGCGCGCCGCTGGTGCGTG<br>CGGAACCGATCCTGACCCGCGAACAGCTGGAAGCGCTCCGTGCCGAACTGGTGAAAACT<br>AGCCGCGCCAAACCGGCGGTATCAACCCCGTCACTGCTGCTGCGCGTGCTGTTTTGTGC<br>CGTTTGTGGCGAACCGGCGTACAAATTTGCCGGCGGTGGTCGCAAACACCCGCGCTACC<br>GCTGCCGCAGCATGGGCTTCCCGAAACATTGCGGTAATGGCACCGTGGCCATGGCGGAA<br>TGGGATGCCTTTTGCGAAGAACAGGTGCTGGATCTGCTGGGCGATGCGGAACGCCTGGA<br>AAAAGTGTGGGTTGCCGGTAGCGATAGCGCCGTTGAACTGGCGGAAGTGAACGCCGAAC<br>TGGTGGATTTGACTAGCCTGATTGGCAGCCCGGCGTATCGTGCAGGTAGCCCGCAGCGC<br>GAAGCGCTGGATGCCCGCATTGCGGCCTTGGCCGCACGTCAAGAAGAACTGGAAGGCCT<br>GGAAGCCCGCCCGAGTGGCTGGGAATGGCGCGAAACCGGTCAGCGCTTTGGTGATTGGT<br>GGCGCGAACAAGATACCGCGGCCAAAAATACGTGGCTGCGTAGTATGAACGTGCGTCTG<br>ACCTTTGACGTGCGCGGCGGTCTGACGCGTACCATTGATTTTGGTGATCTGCAAGAGTA<br>CGAACAGCATCTGCGTCTGGGCAGCGTGGTGGAACGCCTGCACACCGGCATGAGC
**RBS**	TCACACAGGAAAG

RFP	ATGGCTTCCTCCGAAGACGTTATCAAAGAGTTCATGCGTTTCAAAGTTCGTATGGAAGG<br>TTCCGTTAACGGTCACGAGTTCGAAATCGAAGGTGAAGGTGAAGGTCGTCCGTACGAAG<br>GTACCCAGACCGCTAAACTGAAAGTTACCAAAGGTGGTCCGCTGCCGTTCGCTTGGGAC<br>ATCCTGTCCCCGCAGTTCCAGTACGGTTCCAAAGCTTACGTTAAACACCCGGCTGACAT<br>CCCGGACTACCTGAAACTGTCCTTCCCGGAAGGTTTCAAATGGGAACGTGTTATGAACT<br>TCGAAGACGGTGGTGTTGTTACCGTTACCCAGGACTCCTCCCTGCAAGACGGTGAGTTC<br>ATCTACAAAGTTAAACTGCGTGGTACCAACTTCCCGTCCGACGGTCCGGTTATGCAGAA<br>AAAAACCATGGGTTGGGAAGCTTCCACCGAACGTATGTACCCGGAAGACGGTGCTCTGA<br>AAGGTGAAATCAAAATGCGTCTGAAACTGAAAGACGGTGGTCACTACGACGCTGAAGTT<br>AAAACCACCTACATGGCTAAAAAACCGGTTCAGCTGCCGGGTGCTTACAAAACCGACAT
GFP	ATGCGTAAAGGAGAAGAACTTTTCACTGGAGTTGTCCCAATTCTTGTTGAATTAGATGGTG<br>ATGTTAATGGGCACAAATTTTCTGTCAGTGGAGAGGGTGAAGGTGATGCAACATACGGAAA<br>ACTTACCCTTAAATTTATTTGCACTACTGGAAAACTACCTGTTCCATGGCCAACACTTGTC<br>ACTACTTTCGGTTATGGTGTTCAATGCTTTGCGAGATACCCAGATCATATGAAACAGCATG<br>ACTTTTTCAAGAGTGCCATGCCCGAAGGTTATGTACAGGAAAGAACTATATTTTTCAAAGA<br>TGACGGGAACTACAAGACACGTGCTGAAGTCAAGTTTGAAGGTGATACCCTTGTTAATAGA<br>ATCGAGTTAAAAGGTATTGATTTTAAAGAAGATGGAAACATTCTTGGACACAAATTGGAAT<br>ACAACTATAACTCACACAATGTATACATCATGGCAGACAAACAAAAGAATGGAATCAAAGT<br>TAACTTCAAAATTAGACACAACATTGAAGATGGAAGCGTTCAACTAGCAGACCATTATCAA<br>CAAAATACTCCAATTGGCGATGGCCCTGTCCTTTTACCAGACAACCATTACCTGTCCACAC<br>AATCTGCCCTTTCGAAAGATCCCAACGAAAAGAGAGACCACATGGTCCTTCTTGAGTTTGT<br>AACAGCTGCTGGGATTACACATGGCATGGATGAACTATACAAATAATAA
T14	TCACACTGGCTCACCTTCGGGTGGGCCTTTCTGCGTTTATATACTAGAGAGAGAATATAAA<br>AAGCCAGATTATTAATCCGGCTTTTTTATTATTT
attP	GGGTTTGTACCGTACACCACTGAGACCGCGGTGGTTGACCAGACAAACCACGA
attB	GCCCGGATGATCCTGACGACGGAGACCGCCGTCGTCGACAAGCCGGCCGA
attL	GGGTTTGTACCGTACACCACTGAGACCGCCGTCGTCGACAAGCCGGCCGA
P_{tet}	TCCCTATCAGTGATAGAGATTGACATCCCTATCAGTGATAGAGATACTGAGCAC
P_{rhaB}	CCACAATTCAGCAAATTGTGAACATCATCACGTTCATCTTTCCCTGGTTGCCAATGGCCCA<br>TTTTCCTGTCAGTAACGAGAAGGTCGCGTATTCAGGCGCTTTTTAGACTGGTCGTAATGAA
FourU	GGACAAGCAATGCTTGCCTTGAATAGTAACTTTTGAATAGTGATTCAGGAGG

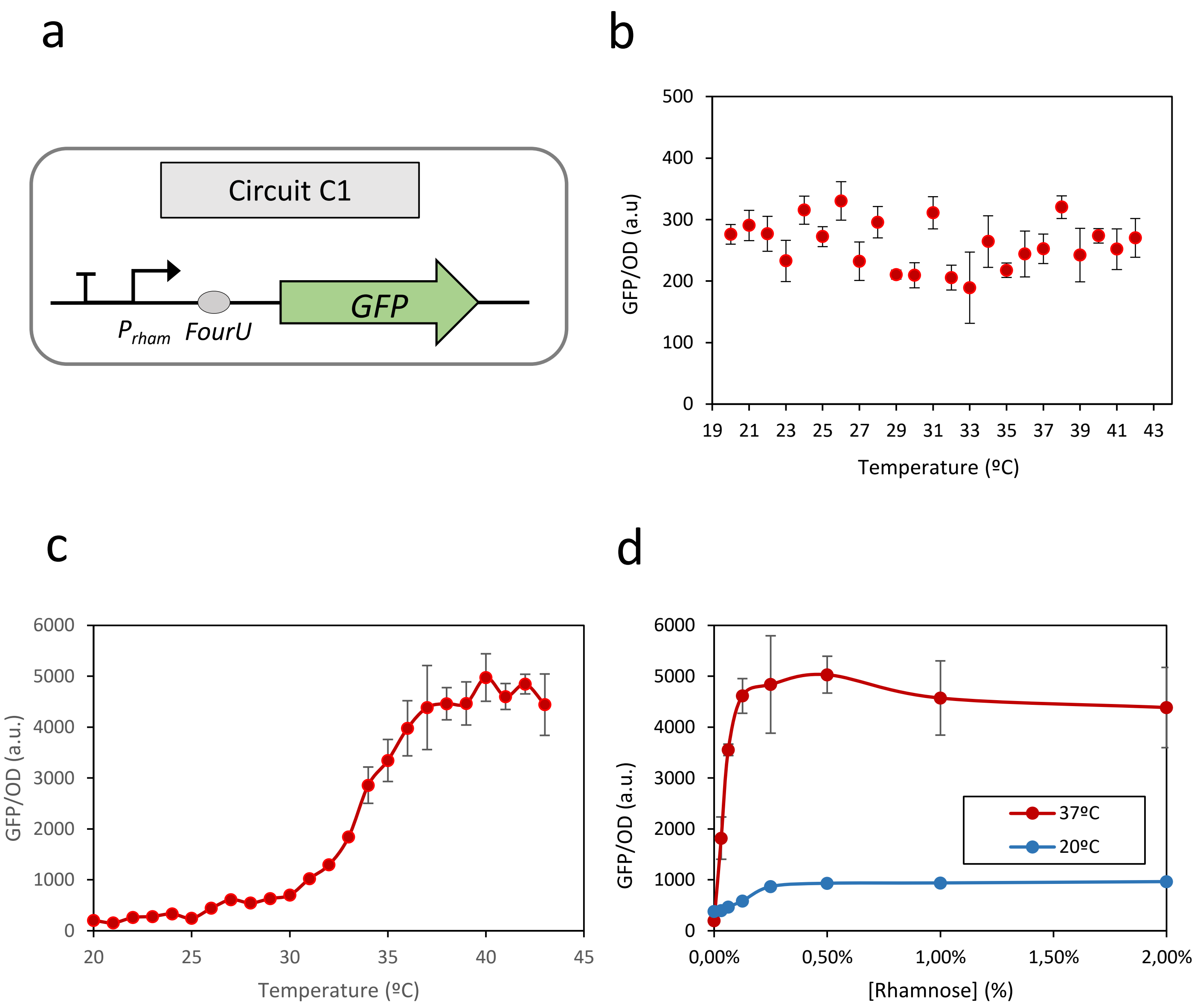

Figure S1. a. Minimal circuit used to characterize the effect of promoter activity on FourU performance. **b.** GFP levels at 0% rhamnose. At this minimal promoter activity, no temperature-dependent GFP expression was observed. **c.** GFP expression at 2% rhamnose. At this higher promoter activity, a clear temperature dependence of GFP expression was observed. **d.** GFP expression at different rhamnose concentrations at low (20 °C) and high (37 °C) temperatures. Error bars represent the standard deviation from three independent experiments.

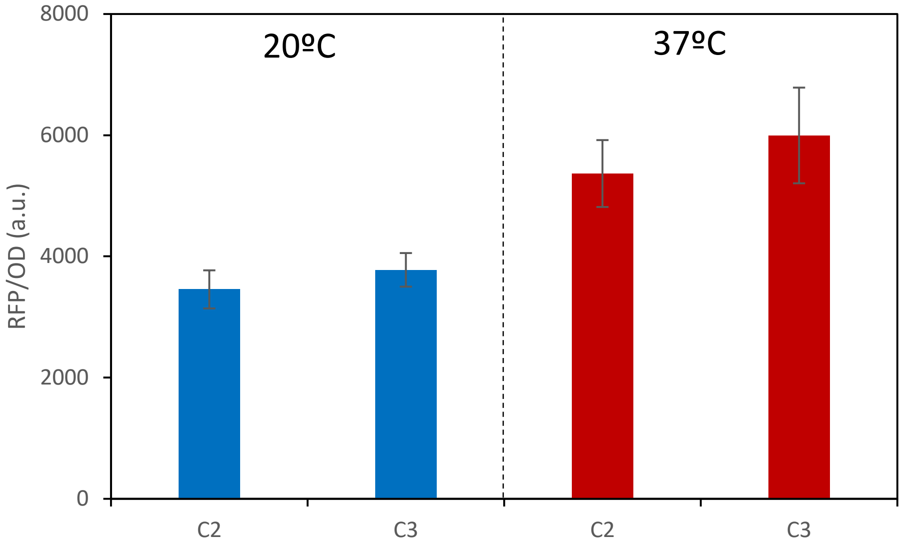

Figure S2. RFP fluorescence levels measured in circuits C2 and C3 at 20 °C (blue bars) and 37 °C (red bars). Welch's t-test yielded p-values of 0.259 at 20 °C and 0.329 at 37 °C, indicating that although RFP fluorescence is affected by temperature, there is no statistically significant difference between the fluorescence levels measured in circuits C2 and C3 at either temperature. Error bars represent the standard deviation from three independent experiments.

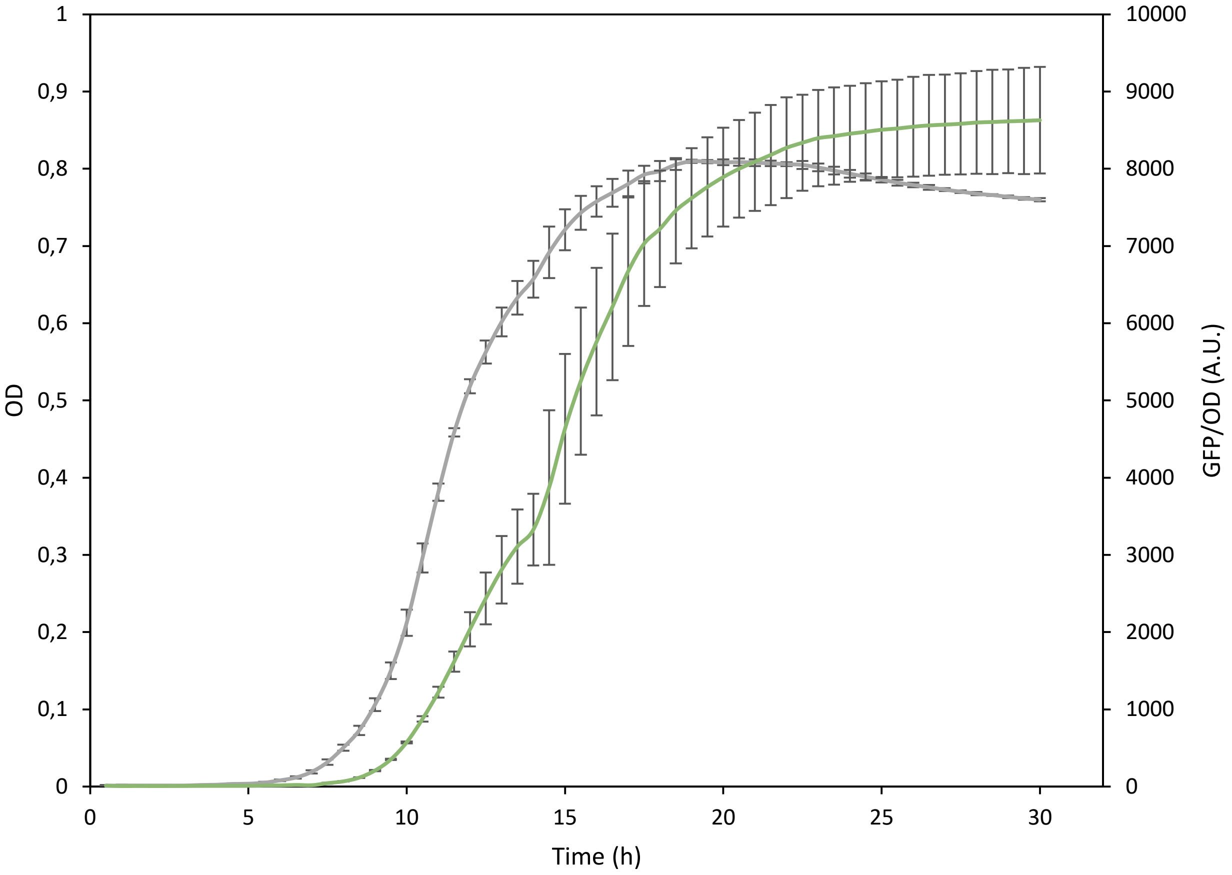

Figure S3. Time evolution of optical density (OD, black line, right vertical axis) and GFP levels normalized to OD (green line, left vertical axis) in circuit C2 at 37 °C. Data represent the mean of three independent experiments. Error bars indicate the standard deviation of the three independent experiments.

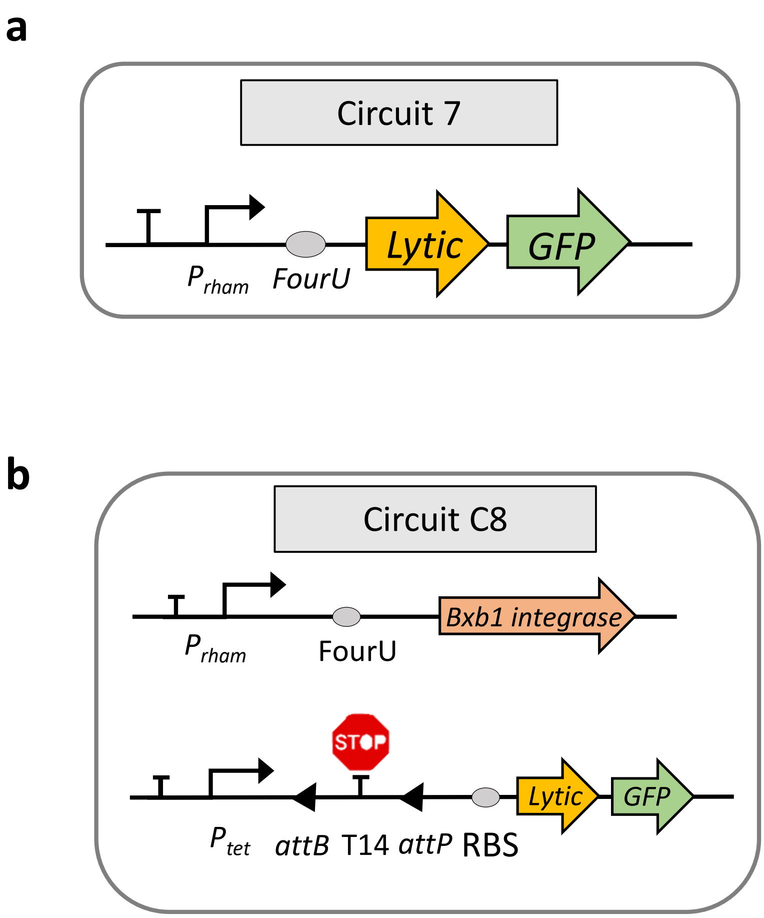

Figure S4: Schematic representation of the genetic architecture of the circuits used for the expression of the lytic gene in response to a temperature increase. Circuit 7 implements temperature-dependent expression of the lytic gene without the use of Bxb1, while Circuit C8 modulates the temperature-dependent expression of the lytic gene through Bxb1.
